# A scoping review of inequities in access to organ transplant in the United States

**DOI:** 10.1186/s12939-021-01616-x

**Published:** 2022-02-12

**Authors:** Christine Park, Mandisa-Maia Jones, Samantha Kaplan, Felicitas L. Koller, Julius M. Wilder, L. Ebony Boulware, Lisa M. McElroy

**Affiliations:** 1 Division of Abdominal Transplant, Department of Surgery, Duke University School of Medicine, Durham, NC USA; 2 Division of Cardiac Anesthesiology, Department of Anesthesiology, Weil Cornell Medicine, New York, NY USA; 3 Medical Center Library and Archives, Duke University School of Medicine, Durham, NC USA; 4 Division of Abdominal Transplant, Department of Surgery, University of Mississippi School of Medicine, Jackson, MS USA; 5 Division of Gastroenterology, Department of Medicine, Duke University School of Medicine, Durham, NC USA; 6 Division of General Internal Medicine, Department of Medicine, Duke University School of Medicine, Durham, NC USA

**Keywords:** Organ transplant, Inequities, Disparities, Access

## Abstract

**Background:**

Organ transplant is the preferred treatment for end-stage organ disease, yet the majority of patients with end-stage organ disease are never placed on the transplant waiting list. Limited access to the transplant waiting list combined with the scarcity of the organ pool result in over 100,000 deaths annually in the United States. Patients face unique barriers to referral and acceptance for organ transplant based on social determinants of health, and patients from disenfranchised groups suffer from disproportionately lower rates of transplantation. Our objective was to review the literature describing disparities in access to organ transplantation based on social determinants of health to integrate the existing knowledge and guide future research.

**Methods:**

We conducted a scoping review of the literature reporting disparities in access to heart, lung, liver, pancreas and kidney transplantation based on social determinants of health (race, income, education, geography, insurance status, health literacy and engagement). Included studies were categorized based on steps along the transplant care continuum: referral for transplant, transplant evaluation and selection, living donor identification/evaluation, and waitlist outcomes.

**Results:**

Our search generated 16,643 studies, of which 227 were included in our final review. Of these, 34 focused on disparities in referral for transplantation among patients with chronic organ disease, 82 on transplant selection processes, 50 on living donors, and 61 on waitlist management. In total, 15 studies involved the thoracic organs (heart, lung), 209 involved the abdominal organs (kidney, liver, pancreas), and three involved multiple organs. Racial and ethnic minorities, women, and patients in lower socioeconomic status groups were less likely to be referred, evaluated, and added to the waiting list for organ transplant. The quality of the data describing these disparities across the transplant literature was variable and overwhelmingly focused on kidney transplant.

**Conclusions:**

This review contextualizes the quality of the data, identifies seminal work by organ, and reports gaps in the literature where future research on disparities in organ transplantation should focus. Future work should investigate the association of social determinants of health with access to the organ transplant waiting list, with a focus on prospective analyses that assess interventions to improve health equity.

**Supplementary Information:**

The online version contains supplementary material available at 10.1186/s12939-021-01616-x.

## Background

Transplantation has been a durable treatment option for patients with end-stage organ disease for over 40 years, with over 30,000 organ transplants performed in the United States (US) in 2019 [1]. Disparities in transplantation affect every organ and span the continuum of transplant care, from referral to postoperative outcomes. Yet, despite several decades of research on disparities in organ transplant, causal mechanisms between social determinants of health and inequities in access to transplant have not been clearly established. It is also unclear if previously developed interventions have resulted in sustained reduction in disparities for disenfranchised groups. We performed a scoping review of the literature for US-based studies that described disparities or identified sources of health inequities along the continuum of transplant care. We hypothesized that most research describing disparities and health inequities in transplant is (1) focused on patients after addition to the organ transplant waitlist, and (2) focused on kidney transplant. We also hypothesized that most research aimed at understanding the influence of social determinants of health on access to transplant is limited to race, gender and insurance status.

## Methods

The research question we aimed to address in this scoping review was: How many US-based studies have quantified disparities or identified sources of health inequities in adults during the transplant selection process (referral, evaluation and selection, living donation, waitlist)? We chose a scoping review due to the broad scope and our intended purpose of identifying the types of evidence available in this area and the gaps in the knowledge base [2].

A medical librarian with expertise in systematic searching (SK) composed a search utilizing a combination of subject headings and keywords to represent the concepts of socioeconomic status, race, organ disease or failure, organ transplantation, and delivery of health care. The databases used were MEDLINE via PubMed, Embase via Elsevier, the Cumulative Index to Nursing and Allied Health Literature Complete via EBSCO, and the Web of Science Core Collection via Clarivate, with a search from journal inception until November 27, 2019. A search update was run on April 20, 2021.

Our inclusion criteria consisted of 1) United States based studies available in English of adults during the selection process or on the waitlist for kidney, liver, pancreas, heart or lung transplant and 2) studies that associate one or more social determinants of health with transplant selection process or waitlist outcomes. We excluded studies that were based outside of the US, not available in English, studies not reporting primary or secondary data (e.g. editorials, opinion pieces and review articles), studies focused on assessment of deceased donor organs, and studies focused on disparities related to a single diagnosis (e.g. sickle cell disease).

All references were compiled in EndNote and imported into Covidence systematic review software (Veritas Health Innovation, Melbourne, Australia. Available at www.covidence.org) for deduplication and screening. When databases allowed for it, non-human studies, editorials, case reports, and conference abstracts were removed. All search strategies are available in the appendix (Additional file 1).

Studies were screened by five investigators (CP, JW, FK, MJ, LM) after an initial study team meeting to discuss the purpose of the review and define the inclusion and exclusion criteria. Full texts were screened by three authors (CP, MJ, LM). Disagreements in screening and were resolved via consensus after discussion.

## Results

**Fig. 1:**
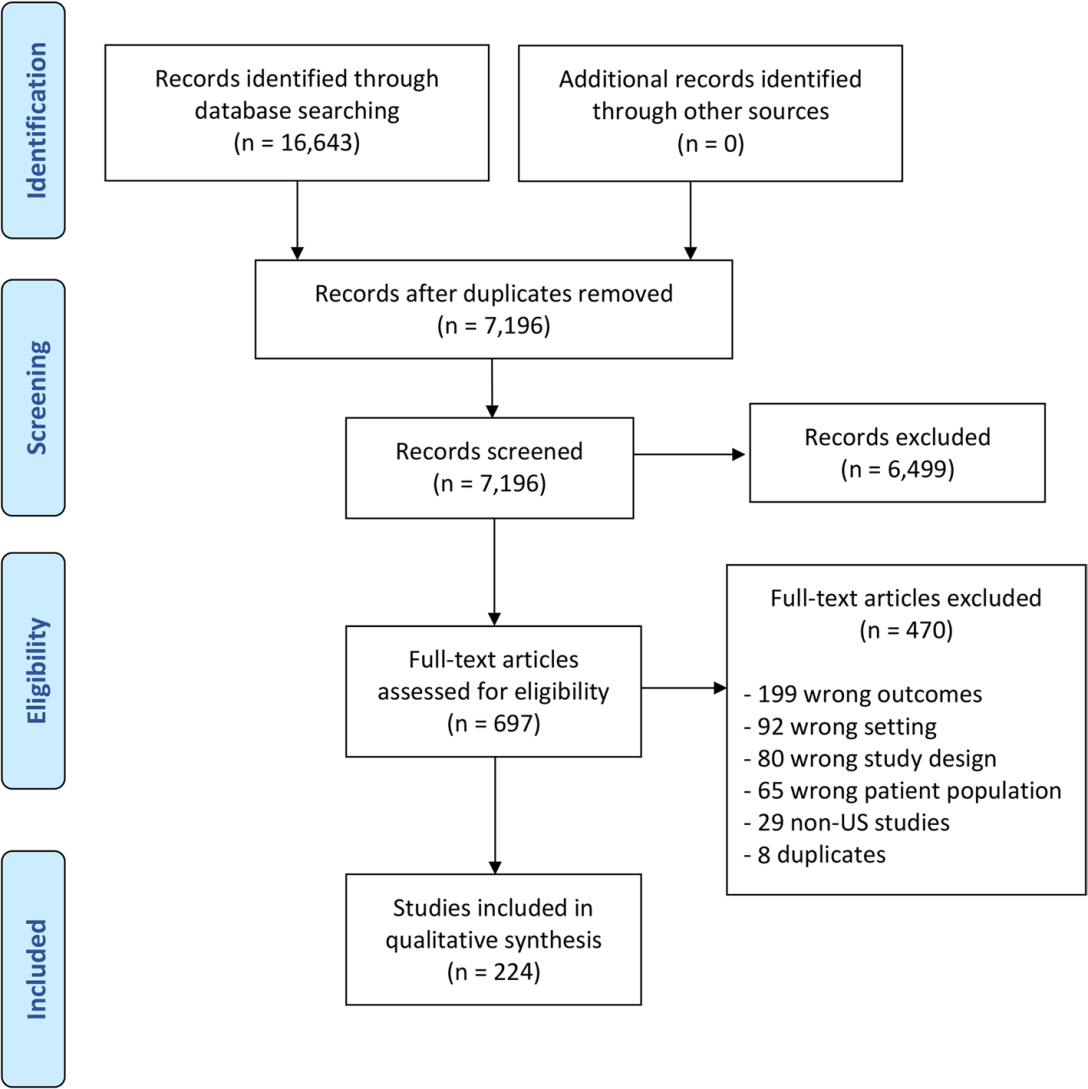
PRISMA diagram outlining the selection process for identifying studies included in the final cohort

In total, 227 studies were identified (Fig. 1), all of which were all performed in the US and published between the years 1992 and 2021. Of these, 35 focused on disparities in referral for transplantation among patients with chronic organ disease, 82 focused on transplant selection processes, 50 on living donors, and 60 on waitlist management; 15 studies involved the thoracic organs (heart, lung), 209 involved the abdominal organs (kidney, liver, pancreas), and three involved both thoracic and abdominal organs (Table 1). There was a total of 13 clinical trials, 8 of which examined racial equity in living donor kidney transplant. Fifteen studies used national transplant registry data (Scientific Registry of Transplant Recipients (SRTR) or United States Renal Data System (USRDS).

**Table 1:** Summary of the included studies stratified by study category and organ types

	Referral	Evaluation and listing	Living donor	Waitlist outcomes	Total
**Mixed solid organs**					
Prospective	–	–	–	–	–
Retrospective	–	3	–	–	3
Clinician survey	–	–	–	–	–
Patient survey	–	–	–	–	–
**Thoracic organs**					
Heart	2	5	–	8	**15**
Prospective	–	1	–	–	1
Retrospective	2	3	–	8	13
Clinician survey	–	1	–	–	1
Patient survey	–	–	–	–	–
Lung	–	–	–	–	**–**
**Abdominal organs**					
Kidney	29	61	49	24	**163**
Prospective	6	9	17	1	33
Retrospective	8	39	17	20	84
Clinician survey	6	3	3	–	12
Patient survey	9	10	12	3	34
Liver	3	11	3	28	**45**
Prospective	–	2	–	2	4
Retrospective	3	8	3	25	39
Clinician survey	–	–	–	–	–
Patient survey	–	1	–	1	2
Pancreas	–	2	–	–	**2**
Prospective	–	–	–	–	–
Retrospective	–	2	–	–	2
Clinician survey	–	–	–	–	–
Patient survey	–	–	–	–	–
**Total**	34	82	52	60	**228**

### Referral of patients with chronic organ disease for transplantation

#### Thoracic

##### Two studies examined referral of patients with heart failure for advanced therapies, including transplantation

A retrospective analysis of the National Inpatient Sample by Thakkar et al. found a lower rate of device utilization and referral for heart transplantation among Medicaid recipients compared to privately-insured patients [3]. Breathett et al. expanded on this association with a qualitative study of transplant clinicians which demonstrated significant reliance on perceptions about the adequacy of social support for women as part of overall candidacy for advanced heart therapies. Specifically, children were perceived as liabilities for women, and family dynamics and finances were perceived to be of greater importance when spouses were deemed to be inadequate supports for the women [4].

#### Abdominal

##### Kidney

***Two clinical trials have tested educational interventions to improve equity in referral for kidney transplant***

The Reducing Disparities in Access to kidNey Transplantation (RaDIANT) Community Study was a dialysis facility-based, randomized clinical trial conducted in 2014 to test the effectiveness of a multicomponent intervention aimed at increasing referral for kidney transplantation in Black patients. The intervention consisted of education and outreach activities for dialysis facility leadership, staff, and patients [5, 6]. The intervention increased the proportion of patients referred for transplant at 12 months (adjusted mean difference of 7.3%) and the difference between intervention and control facilities was greater among Black vs White patients (adjusted mean difference of 6.4% vs 3.4%) [7].

The Explore Transplant at Home study was a prospective parallel-arm, randomized controlled trial that compared the effect of educational interventions on patients’ knowledge of transplant. The intervention arms were: (1) transplantation education provided in dialysis centers only (standard of care), (2) a patient-guided Explore Transplant at Home program, and (3) the Explore Transplant at Home program facilitated by a telephonic educator [8]. Patients’ knowledge of transplant as assessed by a post-intervention survey using the transplantation knowledge scale showed increased likelihood of transplant evaluation initiation versus control groups (38% vs 24%, *P* = .006) [9]. The number of discrete, new steps taken by those in the patient-guided program group was also higher than in the control group (incident rate ratio, 1.21; 95% confidence interval [CI], 1.01–1.47; *P* = .04), but there was no difference between the educator-guided and control groups [10].

***Three retrospective analyses of national data have described inequities in referral for kidney transplant***

Both studies used the USRDS to examine the timing of transplant referral. Inclusion of the race coefficient in the Chronic Kidney Disease Epidemiology Collaboration (CKD-EPI) study’s estimation of glomerular filtration rate (GFR) was examined by Zelnick et al. with the goal of determining the impact on eligibility for transplant. The CKD-EPI formula was found to overestimate GFR by a mean of 3.1 mL/min/1.73 m^2^ (95% CI 2.2–3.9 mL/min/1.73 m^2^; *P* < .001) [11]. This error in estimate was associated with a 35% (95% CI 29–41%) higher risk of achieving an eGFR less than 20 mL/min/1.73 m^2^ and a shorter median time to transplant eligibility by 1.9 years. Kucirka et al. found that a significant proportion of patients had not received information about the option of transplant at the time of dialysis initiation, most commonly due to lack of clinician assessment. Notably, patients with advanced age, obesity, public or no insurance and at for-profit centers were less likely to be assessed [12]. Despite being less likely to receive transplant education, Black and Hispanic patients had a lower prevalence of medical barriers to transplantation than White patients at the time of dialysis initiation [13].

***Five regional/single-center studies have described inequities in referral for kidney transplant***

Lack of formal education, minority race, Hispanic ethnicity and female gender were all negatively associated with referral to a transplant center [14–17]. A single center study of the impact of the race coefficient in the CKD-EPI eGFR equation showed one-third of patients could be reclassified to a more severe CKD stage if the race multiplier were removed, and 3.1% of patients would newly qualify for accumulating kidney transplant priority [18].

***Six studies surveyed clinicians for their perceptions of inequities in referral for kidney transplant***

Referring nephrologists and dialysis staff both demonstrated knowledge deficiencies, including lack of experience with or recognition of gender and racial disparities in access to kidney transplant [19, 20]. Nephrologists reported being less likely to believe that transplantation prolongs survival relative to dialysis for Black patients in comparison to White patients and differences in referral for renal transplantation arise from differences in patients’ preferences rather than from problems with communication, trust, or racial bias that may relate to physicians’ actions or inaction [21]. Despite acknowledgment of the importance of education, nephrologists treating predominantly Black, elderly, and Medicaid-insured patients have reported insufficient time as the primary barrier to transplant education [22]. Furthermore, surveyed clinicians reported increased referral of patients with high school or greater education. Patients less likely to be referred included homemakers and other unemployed patients compared with employed patients [23]. Low socioeconomic status and age were identified as the primary drivers of racial disparities in rates of referral. Dialysis center characteristics associated with lower odds of recommending transplant included those in rural locations, with for-profit ownership, and with greater percentages of uninsured patients and/or more patients older than age 65 [24].

***Nine studies surveyed patients for their perceptions of inequities in referral for kidney transplant***

Black patients and women reported lower rates of referral and later referral for transplantation, poor treatment by medical professionals, and poor education about renal disease and treatment options [25–27]. Concerns about pursuing kidney transplant included increased medication burden, fear of surgery, and fear of organ rejection [28]. Barriers to initiating transplant evaluation included confusion about diagnosis, lack of transplant knowledge, financial burdens, transportation, scheduling, emotional overload of chronic illness, medical mistrust, experiences with discrimination, and perceived racism [29, 30]. Lack of knowledge about transplantation and insufficient patient-clinician communication were cited as persistent barriers to transplant, even in a setting of strong social support, high self-reported interest, and adequate insurance coverage [25, 31–33].

##### Liver

***Three studies have described inequities in referral for liver transplant***

In a Veterans Administration (VA)-based study, liver transplantation was considered for only 21% of patients satisfying referral guidelines from the American Association for the Study of Liver Disease. Negative determinants were older age, presence of alcoholic liver disease, and Black race [34]. Two additional studies that used the SRTR data reported that patients of female gender, Black race, and Medicare insurance were less likely to be referred and evaluated for liver transplantation [35]. Of those referred, Black patients had a higher likelihood of residing closer to the transplant center and having a higher Model for End-Stage Liver Disease (MELD) score at presentation [36].

### Transplant center selection processes

#### Non-organ specific

***Three studies investigated inequities in the transplant selection process for all solid organs***

In a study of all solid organ transplant candidates, patients living in rural areas had a lower rate of waitlisting and transplant despite equivalent outcomes following transplant, suggesting barriers to evaluation and waitlist entry for rural residents with organ failure [37]. Patients with limited reading or math ability and patients with cognitive impairment were also less likely to be listed for transplant and more likely to be removed from listing or to miss prelisting appointments [38]. For all organs except liver, the number of transplants performed among Medicaid beneficiaries was only half of the expected number based on the Medicaid-insured population. Medicaid transplant recipients were listed with more severe organ failure, suggesting a longer time to successfully complete the evaluation process [39].

#### Thoracic

##### Heart

***Five studies (one prospective, three retrospectives, one clinician survey) investigated inequities in the transplant selection process for heart failure patients***

These studies demonstrated that age, gender, income, insurance status and race all influence decision-making for advanced heart failure therapies, with patients who are over age 40, female, and Black less likely to be selected for transplant [40]. Patient adherence and social history both were found to weigh heavily on clinician decision-making [41, 42]. Medicare and Medicaid insurance predicted lower odds of both left ventricular assist device (LVAD) and eligibility for heart transplant [43]. However, Medicaid expansion resulted in a 30% increase in the rate of heart transplant listings for Black patients in early adopter states [44].

#### Abdominal

##### Kidney

***Three clinical trials tested interventions to decrease disparities in completion of the transplant selection process and approval for the kidney transplant waitlist***

A randomized controlled trial that aimed to test the effectiveness of trained navigator assistance in the process of referral to selection committee decision found an increase in waitlisting (primary outcome) and a reduction in time from referral to waitlisting (secondary outcome) [45]. The study did not demonstrate significant difference in the listing rate between patients in the control and intervention groups. Time from referral to waitlisting was 126 days longer for patients in the intervention group, and these patients were 3.3 times more likely than patients in the control group to be waitlisted after 500 days (75% vs 25%; hazard ratio, 3.31; 95% CI 1.20–9.12).

The iChoose Kidney study was a randomized controlled trial to test the clinical efficacy of an electronic application designed to educate patients about the survival benefit of kidney transplantation. The primary outcome was change in knowledge about the survival benefit of kidney transplantation vs dialysis [46]. Change in knowledge as assessed by survey was greater among intervention (1.1 ± 2.0) vs control (0.4 ± 1.8) patients (*P* < .0001). However, access to transplantation was similar [47].

The Allocation System Changes for Equity in Kidney Transplantation (ASCENT) trial is an ongoing randomized controlled effectiveness-implementation study designed to test the effectiveness of a multicomponent intervention to improve access to the early steps of kidney transplantation among dialysis facilities across the United States (US). The intervention consisted of an educational webinar for dialysis medical directors, educational videos for patients and dialysis staff, and a dialysis facility-specific transplantation performance feedback report. There was minimal difference between the intervention and control groups for the primary outcomes (change in proportion of patients waitlisted, and disparity reduction in proportion of patients waitlisted in a dialysis facility after 1 year) [48].

***Five studies prospectively assessed inequities in the kidney transplant selection process***

Both race and gender have been independently associated with decreased likelihood of completing the evaluation process, with significant interaction between race and income as well as gender and income [49, 50]. Black patients were found to be 25% less likely (95% CI 0.60–0.96) to be waitlisted than White patients even after adjusting for medical factors and other social determinants of health. Advanced age, low income, public insurance, comorbidities, and dialysis status also decreased the probability of waitlisting. On the other hand, increased social support and transplant knowledge increased the probability of waitlisting [51]. Failure to complete the selection process was more commonly caused by remaining stationary rather than moving backward in the process or dying [52]. Educational sessions increased the one-year evaluation completion rates (adjusted risk ratio of completion at 1 year = 1.38, 95% CI 1.12–1.71), particularly among Black patients and patients living in nonaffluent neighborhoods [53].

***Twenty-one retrospective studies of national data registries examined disparities in completion of the transplant selection process and approval for the kidney transplant waitlist***

Studies using the United Network for Organ Sharing (UNOS) data have demonstrated low rates of addition to the waitlist for Black and female patients on dialysis [54, 55]. Low rates of preemptive listing and prolonged exposure to prelisting dialysis were associated with Medicare insurance, minority race/ethnicity, low educational attainment, longer distance to transplant center, and residence in distressed communities [56, 57].

Studies using the USRDS data have demonstrated low rates of addition to the waitlist among women, minorities, patients receiving dialysis at facilities located further away from transplant centers, and the elderly [58–60]. Among UNOS regions, racial disparities appear to be more pronounced where Black patients comprise a large proportion of the end stage renal disease (ESRD) population [61]. Factors shown to mitigate these characteristics included education, social support, and employment [62–68]. Interestingly, early referral to a nephrologist has been shown to increase the likelihood of successful completion of the process [69]. State Medicaid coverage seemed to mediate both the incidence of ESRD and insurance-related differences in access [70].

Studies combining the USRDS and the UNOS databases have allowed for examination of the influence of comorbidity profile and dialysis vintage on access to the transplant waitlist. These studies reinforce prior knowledge of decreased likelihood of waitlisting and transplant among minorities, the impoverished, and the elderly [71–73]. Implementation of the kidney allocation system (KAS) in 2014 has reduced inactive waitlisting among minorities and has contributed to reduced racial disparity [74].

***Eighteen retrospective analyses of regional and single-center data have identified disparities in completion of the kidney transplant selection process***

These studies largely reinforced the findings of national analyses: that female gender, advanced age, non-White race, low educational level, low income, and Medicaid insurance are associated with decreased rates of listing for transplant and increased waitlist mortality [75–91]. The extent of patient support networks and provision of enhanced educational sessions at the time of referral for renal transplant are associated with increased completion of the evaluation process [92].

***Three studies surveyed clinicians for their perceptions of inequities in the kidney transplant selection process***

Clinician-reported impressions of patient barriers to transplant included negative personal behaviors such as individual irresponsibility and lack of self-management of comorbidities as well as limited income, transportation difficulties, insurance issues, and limited/lack of social support. Furthermore, clinicians reported believing that patients are largely responsible for the development of ESRD, and increased social responsibility was needed to improve poor health status and disparities in kidney transplantation rates [93]. When assessing personal responsibility and self-management as part of candidacy determination, clinicians included assessing adherence but used proxies such as the number of missed hemodialysis sessions, therapeutic drug levels, and the Simplified Medication Adherence Questionnaire [94]. Educational barriers were also identified by providers which included difficulty accessing information about transplantation, limited knowledge of transplantation, lack of formal education with associated literacy and/or reading comprehension challenges. Providers also identified systemic barriers such as scheduling difficulties, late or delayed referrals for transplant, poor patient-staff communication, and limited access to transplant coordinators [95].

***Ten studies reported patient perspectives on barriers to completion of the kidney transplant selection process***

Surveyed patients echo the findings of national analyses: that early transplant awareness promotes engagement in the transplant subsequent waitlisting and the impact is more pronounced in White vs Black patients [96]. Evaluation completion is less likely in patients who were previously evaluated at a different transplant center as well as patients already on dialysis at the time of referral with the financial burden of kidney transplantation being a prominent deterrent [97].

Minority patients also reported low rates of transplant education and a high level of misinformation about the different stages in the evaluation process, with common misbeliefs being that dialysis must precede transplantation and that transplantation is only available as a last resort [98–101]. A significant number of patients undergoing evaluation for transplant remained unaware of their listing status, including mistakenly believing they were already on the waitlist [102].

The reasons for patients declining transplant were commonly due to negative personal experience with previous transplant and/or hearing about another patient’s negative experience [103]. Other prominent patient-reported barriers to transplant included financial concerns and poor communication [98, 104]. Barriers were most prominent in racial/ethnic minorities and people with low income. Black patients were more likely than White patients to report racial discrimination, and women were more likely than men to report gender discrimination during the evaluation and selection process [105].

##### Liver

***Two studies prospectively assessed inequities in the liver transplant selection process***

A prospective assessment of liver candidate literacy revealed that low health literacy was independently associated with not being waitlisted for transplant, although the effect was attenuated by strong social support [106]. A national assessment of transplant center websites examined disparities in access to the waitlist using the Website Clear Communication Index and demonstrated that each point increase in the index score (signaling improved communication) was associated with a 0.2% increase in waitlisting of low educational attainment patients [107].

***Three retrospective analyses of national data described inequities in the liver transplant selection process***

All studies used the UNOS database and found increased rates of medical unsuitability for transplantation, death during the evaluation process, and waitlist mortality among racial minority patients [108–110]. Although time spent on waitlists was similar, Black race negatively affected outcomes while awaiting liver transplant.

***Five regional or single-center studies examined inequities in the liver transplant selection process***

These studies echoed the findings of larger studies, demonstrating association of minority status with lower odds of being listed and a longer time to selection committee decision compared to other patients, even after adjusting for socioeconomic factors [111, 112]. Black patients with hepatocellular carcinoma were less likely to undergo liver transplantation than White patients, and substance abuse was more frequently cited as the reason Black patients within the Milan criteria failed to undergo transplantation compared to White patients [113]. Successful listing for liver transplant was also associated with private insurance and access to a local gastroenterologist [114]. A study of the effects of telehealth on the liver transplant selection process found a reduction in the time from referral to evaluation and listing, but not to transplantation [115].

***A single study reported patient perspectives about inequities in the liver transplant selection process***

All patients reported wanting liver transplant if recommended. Compared with non-listed patients, waitlisted patients had attained higher education levels and were more likely to be privately-insured [116]. Non-listed patients were significantly less likely to have discussed liver transplant with their physician, be referred to a transplant center, or be approached by the transplant center once referred.

##### Pancreas

***Two retrospective analyses of national data have described inequities in the pancreas transplant selection process***

A study using the USRDS data revealed that White patients received 92% of all simultaneous pancreas-kidney transplants (SPKT) [117]. Lack of private insurance and unemployment status were associated with lower transplant rates. Following initiation of Medicare coverage for SPKT in 1999, Black and Hispanic patients had almost 30% lower SPKT registration rates than their White counterparts, and the disparity was more substantial for non-White patients with primary Medicare insurance than those with private insurance [118].

### Living donor identification and evaluation

#### Abdominal

##### Kidney

***Eleven clinical trials have tested interventions to reduce disparities in living donor kidney transplant (LDKT): six trials focused on educational interventions, three focused on enhanced support for patients identifying living donors, and two focused on changing care delivery for potential living donors. Three studies were ongoing at the time of our query***

*Educational Interventions:*

The Enhancing Living Donor Kidney Transplant Education (ELITE) Study was a cluster randomized trial assessing the ability of an educational intervention to improve knowledge of LDKT. Five hundred potential transplant candidates were cluster-randomized to receive either: (1) usual standard-of-care transplant education or (2) intensive education based upon the Explore Transplant educational materials [119]. The intensive education resulted in higher knowledge compared with usual care (12.7 vs 11.7; *P* = .0008) and increased willingness to take steps toward LDKT. However, there were no differences in post-evaluation readiness for LDKT [120].

A multisite randomized controlled trial evaluated the efficacy of exposure to a bilingual, culturally-targeted website (titled *Infórmate*) for increasing Hispanic patients’ knowledge about LDKT. Website content (including images, telenovela scripts, and messages) was informed by focus groups composed of adult Hispanic kidney transplant recipients, living kidney donors, dialysis patients, and the Hispanic community [121]. Following implementation, website exposure was associated with a mean increase of 22% in knowledge of LDKT which was sustained at 3 weeks post-exposure, compared with control scores that increased by 12% (*P* = .0001). Website exposure was also associated with a 10% greater knowledge score at three-week follow-up (*P* < .0001), and 93% of patients reported a plan to return to *Infórmate* in the future [122]. A separate pretest/posttest intervention study was conducted among adult Hispanic patients undergoing dialysis at five dialysis centers and showed website exposure was associated with a mean 17% same-day knowledge score increase between pretest and posttest (*P* < .001) that was sustained at 3 weeks. Most participants (95%) “agreed” or “strongly agreed” that they would recommend the website to other Hispanic patients [123].

A multicenter randomized controlled trial tested the effectiveness of a revised Living ACTS (About Choices in Transplantation and Sharing) intervention to increase knowledge of LDKT and willingness to discuss LDKT with family members. The web-based intervention Living ACTS was developed for Black patients with kidney disease and included five educational modules: Introduction, Benefits and Risks, The Kidney Transplant Process, Identifying a Potential Kidney Donor, and ACT Now (which encourages communication with friends and family about transplantation) [124]. Intervention participants demonstrated a significantly greater increase in knowledge of LDKT and greater willingness to talk to their families about LDKT than did control participants. However, the effect of the intervention was not sustained at six-month follow-up [125].

A parallel group, two-arm randomized controlled trial is testing an educational and behavioral intervention designed to increase receipt of LDKT among Black transplant candidates [126]. Candidates on the waitlist are randomly assigned to one of two conditions: (1) a control group that will receive usual care, or (2) an intervention group that will receive Destination Transplant, a nine-month intervention that includes an in-person group-based education session, postcards at monthly intervals, and a follow-up phone call from a transplant educator. At baseline and during 18 months of follow-up, demographic, clinical, and other variables are to be collected, such as transplant derailers (factors that might be sources of delay, difficulty, or challenge to pursuing transplant), transplant knowledge, health literacy, small steps taken to pursue LDKT, readiness for LDKT, decisional balance and self-efficacy measures, decisional conflict, family support, availability of potential living donors, and general health status.

Your Path to Transplant is a randomized controlled trial of a computer-based education intervention to increase LDKT. The education intervention consists of individually-tailored telephonic coaching sessions, feedback reports, video- and print-based transplant education resources, and assistance with reducing any known socioeconomic barriers to LDKT [127].

Talking About Live Kidney Donation (TALK) is a two-phase mixed-methods study to design and test culturally-sensitive interventions to improve patients’ consideration of LDKT. Phase 1 involved the development of written and audiovisual educational materials and an accompanying social worker intervention to encourage patients’ engagement in consideration of LDKT [128]. Phase 2 is a randomized controlled trial where patients with CKD are assigned to receive: (1) usual care by their nephrologists, (2) usual care plus the educational materials, or (3) usual care plus the educational materials and social worker intervention. The primary outcome is self-reported rates of consideration of LDKT, including family and patient-physician discussions and identification of a living kidney transplant donor.

*Enhanced support for patients identifying living donors*

House Calls is an intervention to directly engage the social network of CKD patients in LDKT education. The intervention consisted of a single 60 to 90-min session delivered to the patient and their social network by health educators in the patient’s home. The randomized trial had three intervention arms in which health educators delivered an intervention to: (1) the patient and his/her guests in the patient’s home (House Calls arm [HC]), (2) clusters of patients and their guests in the transplant center (Group-Based arm [GB]), or (3) the individual patient alone in the transplant center (Individual Counseling arm [IC]). At the two-year endpoint, HC patients were more likely than GB and IC patients to have at least one donor inquiry (82% vs 61% vs 47%; *P* = .001) and evaluation (65% vs 39% vs 27%; *P* < .001). HC patients also were more likely than other patients to have higher knowledge, fewer concerns, and higher willingness to talk to others about donation 6 weeks post-intervention [129]. A subsequent secondary analysis of data collected as part of the House Calls trial assessed LDKT readiness stage, knowledge, concerns, and willingness to talk to others about living donation at 2 years post-intervention. 60% of patients were not considering or not yet ready to pursue LDKT, while only 11% had taken action to talk to family members or friends about the possibility of living kidney donation. Patients in later stages of LDKT readiness (i.e., had talked to others about donation or were preparing to do so) had significantly more knowledge (*P* < .001), fewer concerns (*P* = .002), and more willingness (*P* = .001) to talk to others about living donation than those in earlier readiness stages [130].

The Providing Resources to Enhance African American Patients’ Readiness to Make Decisions about Kidney Disease (PREPARED) Study was a six-month randomized controlled trial of Black patients with ESRD who had recently initiated in-center hemodialysis. Participants were randomly assigned to receive: (1) usual dialysis care in the dialysis facility, (2) informational decision aids (i.e., a video and a book describing LDKT and other forms of renal replacement therapy, referred to as “PREPARED information”), or (3) the PREPARED information plus a living kidney donor financial assistance program [131]. In total, 62% of participants reported that interventions helped their decision-making about renal replacement treatments, but there were no statistically significant improvements in LDKT actions among groups at 6 months and no participants utilized the living donor financial assistance benefit [132].

The Talking about Living Kidney Donation Support (TALKS) study in an ongoing trial designed to evaluate the effectiveness of three interventions by conducting a randomized controlled trial in which patients on the deceased donor waitlist receive: (1) usual care while on the transplant waitlist, (2) an educational and social worker intervention, or (3) an educational and social worker intervention plus the option of participating in a financial assistance program. The primary outcome of the study measures potential recipients’ live kidney donor activation (a composite rate of live donor inquiries, newly completed live donor evaluations, or live kidney donation) at 1 year [133].

*Changing care delivery for potential living donors*

An ongoing, two-part study to evaluate the effectiveness of a streamlined single-day evaluation process (dubbed KTFT-TALK) is comprised of (1) Kidney Transplant Fast Track (KTFT) aimed at increasing transplant rates), and (2) the Talking About Live Kidney Donation (TALK) educational intervention aimed at increasing LDKT. The KTFT approach involves completing most or all testing on the same day when candidates arrive for their first pre-transplant clinic appointment. Patients were randomly assigned to the TALK intervention after initial study recruitment [134]. Another ongoing trial based on the TALK interventions uses an effectiveness-implementation hybrid design involving pre-post intervention evaluation with matched controls to implement a complex culturally-targeted intervention at two transplant centers in Dallas, Texas and Phoenix, Arizona. The goal of the TALK component here is to evaluate the effect of Northwestern Medicine’s® Hispanic Kidney Transplant Program’s (HKTP) key culturally-targeted components (outreach, communication, education) on Hispanic LDKT rates over 5 years [135].

***Eleven retrospective studies of national databases described inequities in LDKT***

These studies have demonstrated lower rates of LDKT among racial/ethnic minority patients [136–140], including preemptive transplant from living donors [141], women [137, 142], and patients with low education and SES level [137, 140, 143, 144]. At the transplant-center level, increased racial disparity has been associated with higher percentages of Black candidates, preemptively listed candidates and low rates of LDKT [145]. Nondirected living donation specifically has been shown to cluster at certain US-based centers with a consistently low rate of receipt by Black patients [146].

***Six regional and single-center retrospective studies investigated disparities in LDKT***

The findings echoed those of larger studies, that neither interest in nor pursuit of LDKT were associated with knowledge, health literacy, or medical mistrust [147]. More Black patients than others initiated dialysis without prior care for CKD; these patients experienced longer time to transplant in the absence of nephrology care [148]. Ineligible recipients were also more commonly Black patients [149–152].

***Two studies reported clinician perceptions of inequities in LDKT***

Although we found no surveys of clinician opinions about disparities in LDKT, the Live Donor Community of Practice within the American Society of Transplantation held a Consensus Conference on Best Practices in Live Kidney Donation in June 2014. Following the conference, the committee suggested several system-level interventions to improve equity in LDKT. These included removal of financial disincentives to kidney donation, implementation for education programming that is culturally-tailored and community-based, use of transplant liaisons between transplant centers and community nephrology care, and additional research to improve understanding LDKT disparities and LKD differences [153, 154].

A survey of ApoL1 genetic testing practices for living kidney donors was disseminated via email to nephrologists and transplant surgeons at 63 transplant centers in the US that currently have had at least 10 Black living donors per year according to the 2015 UNOS data [155]. A high degree of variability in ApoL1 testing practices was found across transplant centers, potentially reflecting the continued clinical uncertainty of the role that ApoL1 testing has in most potential at-risk donors. The approach taken by most transplant centers consisted of a donor candidate’s self-report of race. Less than 20% of surveyed clinicians expressed concern about ApoL1 testing stigmatizing minorities.

***Eleven studies reported patient perspectives about inequities in LDKT***

Lack of knowledge and misinformation are commonly reported barriers to LDKT among racial and ethnic minority patients. Although patients with ESRD report being willing to accept a kidney from a living donor, they also admit discomfort with asking someone to donate [156]. Donors report having encountered negative responses from others about their desire to donate and also refusal of recipients to accept an LDKT offer [157]. Black donors also reported concerns related to experiences of racial discrimination by healthcare clinicians [158]. Various types of social support have helped donors and recipients navigate the transplant process, including coping strategies. Recipients identified faith as a coping mechanism, while donors identified normalization of donation as their method of coping [104]. Attitudes toward dialysis are moderated by both trust in LDKT and trust in racial equity as related to LDKT [159]. Decisional balance and self-efficacy are important mediators of trust and discomfort among all races and education levels [160].

Women report less interest in receiving LDKT than men, despite being nearly twice as likely as men to receive unsolicited offers for kidney transplant. Women are also less likely than men to have been evaluated for a kidney transplant [161]. However, women make more living donor requests than men, irrespective of race. The barrier to a living donor transplant for most women was not the size of their social support network or lack of requesting a living donor, but rather undefined factors related to the network members themselves [162].

Costs of care and concern for financial strain are commonly reported barriers for donors and recipients. Out-of-pocket expenses were greater for transplant recipients than donors, even though worries about future medical costs were common in both groups [98]. Racial and ethnic minorities in particular expressed unique concerns related to socioeconomic stresses and ways of coping with the stress of donation [158]. Non-English-speaking recipients reported concerns about disqualification of prospective family member donors due to medical issues, inability to miss work, undocumented residency status, inability to support their family if they donate, and declining social support because of illness [163]. This may influence participation in evaluation processes, where consistent attendance has been associated with concerns about finding a living donor (*P* = .038) and higher perceived general knowledge about transplantation (*P* < .001) [97].

##### Liver

***Three studies investigated disparities in access to living donor liver transplant (LDLT)***

National registry data shows that racial/ethnic minorities receive a disproportionately low percentage of LDLTs, and this is thought to be in part due to fewer initial inquiries by potential donors [164]. LDLT is also less likely for recipients who are older, single, divorced, immigrants, and from the lowest income quintile [165]. When surveyed, patients reported preferring to donate and die while the transplant recipient lives, rather than forgo donation and have the potential transplant recipient die of liver failure. Participants’ stated threshold for living donation was a median survival for themselves as donor of 79%, along with a median survival of 55% for recipients with transplantation, before they would agree to donate. Race was the most statistically significant predictor of those thresholds. 81% of respondents believed that the potential donor, not a physician, should have the final say regarding candidacy for living donation [166].

### Waitlist outcomes (including organ selection/utilization)

#### Thoracic

##### Heart

***Eight studies investigated disparities in waitlist outcomes among heart transplant candidates***

Black and Hispanic patients were more likely to be listed with higher urgency (listing status 1A/1B) compared with White patients, and Hispanic patients were at higher risk of waitlist mortality [167]. Black race was found to be significantly associated with longer time to cardiac transplantation [168], lower likelihood of receiving a heart transplant, and greater likelihood of being removed from the waitlist due to worsening health [169, 170]. Women receiving LVAD support also had a reduced chance of heart transplantation and increased risk of waitlist mortality and delisting for worsening clinical status within 2 years post-implantation [171]. Low median household income was associated with an increased risk of readmission and time to first event after LVAD implantation [172], and waitlist mortality was found to be greater among Medicaid beneficiaries [173]. The single study that investigated transplantation as a primary outcome demonstrated no difference by insurance status [174].

#### Abdominal

##### Kidney

***One prospective study described disparities in waitlist outcomes among kidney transplant candidates***

Older age, higher number of comorbidities, transplantation before changes to the KAS, greater religiosity, less social support, and fewer learning activities were each associated with a decreased probability of deceased-donor kidney transplant [175]. Black race, older age, lower income, public insurance status, higher body mass index, dialysis before kidney transplant, not presenting with a potential living donor, religious objection to living-donor transplant, and less transplant knowledge were each associated with a decreased probability of living-donor transplant.

***Twenty retrospective studies of national databases have described disparities in waitlist outcomes among kidney transplant candidates***

National waitlist and transplant rates were comparatively low among minority populations, particularly in high-ESRD incidence donor service areas [176]. Poverty rate, Black race, education, and unemployment in the surrounding community are all significant modifiers of center-level transplant rates [177, 178]. Simulations showed that 30 to 65 waitlist spots or transplant operations per 1000 patients would shift from economically advantaged to disadvantaged persons if socioeconomics no longer influenced organ allocation decisions [179].

Preemptive deceased donor kidney transplant occurs most frequently in patients with private insurance, previous (non-kidney) transplant, and zero-antigen mismatch [180–182]. Implementation of KAS led to an increase in Black and Hispanic patients on the kidney transplant waitlist [183] and an increase in preemptively transplanted minorities [184–186]. However, large differences in the adjusted probability of deceased donor kidney transplantation persisted under KAS, with probability of transplantation significantly associated with organ offer acceptance patterns at transplant centers [187].

Among waitlisted patients, racial and ethnic minorities were more likely to be hospitalized while waitlisted, decreasing the likelihood of being transplanted [188]. Once on the inactive list, White patients were more likely than Hispanic patients to resolve issues for inactivity, resulting in reactivation [189]. Nationally, deceased donor transplantation occurred at a lower rate for Black, Hispanic, Asian/Pacific Islander, and other minority groups compared to White patients [190]. Older patients and Black and Asian recipients also received lower-quality organs relative to young non-Hispanic White patients [191].

Studies examining USRDS data largely echoed the same findings. Adjusted rates of deceased donor kidney transplantation were significantly lower among racial/ethnic minority groups compared to White patients. Differences in time to waitlisting were not as pronounced as differences in time between waitlisting and transplantation. Determinants of delays in time to transplantation differed substantially by racial/ethnic group [192, 193]. A decreased likelihood of deceased donor transplantation was also associated with long travel distances to the transplant center [194]. VA-insured and Medicare/Medicaid-insured patients were less likely to be placed on the waitlist and less likely to receive transplants than patients with private insurance. VA patients with supplemental private insurance had the same likelihood of transplantation as non-VA patients with private insurance [195].

***Three studies reported patient perspectives on barriers to kidney transplant among waitlisted patients***

Most caregivers reported either little-to-none or mild-to-moderate burden, which was greatest among the caregivers of patients who received assistance from someone else (a third party) for activities of daily living (ADLs) [196]. Factors associated with patient-reported likelihood of being waitlisted included Medicare and Medicaid insurance, Black race, community deprivation, and non-US citizen status [197]. Race-related differences in SES, health and functional status, severity of illness, biological factors, existence of contraindications to transplantation, transplant center characteristics, and patients’ attitudes about dialysis and transplantation were predictive factors [198].

##### Liver

***Two single-center prospective cohort examined disparities in waitlist outcomes among patients listed for liver transplant***

Women on the waitlist for liver transplant were significantly more likely to have non liver-related comorbidities, pain medication usage, and infectious complications. Women also had an increased risk of hospitalization, an increase in number of inpatient days within 12 months of listing and a greater risk of dying in the hospital [199, 200].

***Twenty-five retrospective studies of national databases and three of single-center data described disparities in waitlist outcomes among patients listed for liver transplant***

Studies using the UNOS database demonstrated an association between social determinants of health and severity of illness at listing. Education, employment, and private insurance were associated with being changed to a lower priority status and patients with Medicaid as their primary payer had a decreased likelihood of receiving a symptom-based exception [201]. Race, Hispanic ethnicity, insurance status, and lower education predicted delisting [202, 203]. Black race, Hispanic ethnicity, female gender, public insurance, and geographic isolation were associated with increased waitlist mortality and decreased likelihood of receiving a liver transplant [204–223]. The three single-center retrospective studies reinforced the national findings, that male gender is highly correlated with the likelihood of getting a transplant [220], public insurance is associated with increased risk of waitlist mortality, compared with private insurance [224] and the ability to improve waitlist outcomes by pursuing transplant at a second center is also concentrated among candidates with private insurance [225].

***One study reported patient perspectives on barriers to transplant among waitlisted patients***

Appalachian residence was associated with lower access to transplantation after listing for liver transplant. This geographic disparity should be addressed in the current debate over reforming donor liver allocation and patient priority for liver transplant [226].

## Discussion

The most simplistic way to look at disparities in transplant is as a matter of scarcity, i.e., a limited number of deceased donor organs to a patient population with large demand. In order to address this, Congress issued the Final Rule which was enacted in 2000 as a way to have a uniform distribution policy of organs across the transplant waitlist.

When we shift, however, from a goal of equality - all patients on the list having similar access to transplantable organs, to equity-all patients receive the support they need to successfully undergo transplantation- the problem becomes much more complex. Health equity in transplantation is an elusive goal, with multi-level barriers and facilitators along the care continuum. Delayed diagnosis/referral of patients with end-stage organ disease and the complexity of the evaluation process—including requisite contact with several clinicians and nuanced multidimensional risk assessment-- all contribute to difficulties accessing the transplant waitlist. Disparities in access to transplant and drivers of inequitable access have been described for more than 30 years. However, it is unclear what actionable knowledge or progress towards equity in access to transplant has been gained since that time.

Sweeping changes which alter organ allocation to listed patients such as the introduction of the KAS system for kidney allocation or the MELD score have made an impact toward equity on patients listed for kidneys and livers. However, these changes have not simplified the process of gaining access to the waitlist. The complexity of the process and resources required to participate in it are exacerbated for patients who pursue transplant from a living donor.

We conducted a scoping review of the literature to identify studies describing disparities and health inequities in transplant. Our goal was to identify the types of evidence available in this area and to identify gaps in the knowledge of which disparities are inequities. We hypothesized that most research describing disparities and health inequities in transplant is (1) focused on patients after addition to the organ transplant waitlist, and (2) focused on kidney transplant. We also hypothesized that most research aimed at understanding the influence of social determinants of health on access to transplant is limited to race, gender and insurance status. Our review identified 227 publications. Of these, 34 focused on referral of patients with chronic organ disease, 82 focused on the transplant selection process, and 111 focused on patients after addition to the organ transplant waitlist. Only 15 studies involved thoracic transplant, and of the 209 focused on abdominal transplant, 166 focused on kidney transplant. In total there were only 13 clinical trials and 11 prospective studies.

The results of this review bring to light several overarching themes, which appear to be consistent across all organs. Race and gender are the most commonly studied social determinants of health and have a significant impact on their own, as well as powerful interactions with other social determinants of health, in influencing access to transplantation. Other social determinants of health, such as education, insurance status, geographic isolation from a transplant center, and family support, are all strongly associated with disparities. However, evidence gaps remain as to the direct causal pathways between social determinants of health and risk of adverse outcomes.

Patient level barriers are a significant component of inequitable access: knowledge, trust, and communication with caregivers and medical providers are consistently identified determinants of access to transplant-even so much as to help overcome other patient-level barriers, such as income. Serious persistent misinformation and lack of awareness of the benefits of transplant continue to be identified when patients from disenfranchised populations are directly surveyed. Multicomponent interventions have focused on activation of patient behavior based on increased information and the overall results show that well-designed educational interventions will motivate initial but not sustained action, resulting in improved initiation of process but not durable reduction in waitlist disparities.

The role of the primary specialist is critical. In particular, direct survey of clinicians reveals interpersonal bias and both racism and sexism in clinical encounters have been cited by investigators and patients. Some of this may be clinician bias related to engagement with the health system and making optimal use of donated organs. Unfortunately, very little work has examined transplant clinician bias and more research is needed in this area. Recent Center for Medicare and Medicaid changes in how dialysis centers are now getting judged on transplant listing vs referral.

When information from all organs is considered, large gaps remain in the evidence connecting social determinants of health to timely referral, successful evaluation and listing for transplant. Reviewing the literature in detail identified several concerns:

1. The overwhelming majority of health equity research in transplantation has been done in kidney transplant, with far fewer studies in liver or thoracic transplant and only two studies focused on pancreas transplant. This is likely due to the existence of USRDS, which allows for tracking of the management of patients with ESRD from initiation of dialysis through death and/or transplantation, but no similar registry exists for patients with liver disease. Progress towards equity in access to liver transplant will remain hindered by lack of epidemiologic data. In addition, no national data source currently exists that reliably distinguishes patient progress through the transplant selection process.
2. Limited work on referral has demonstrated that although interventions aimed at increasing patient knowledge are generally effective, they are insufficient to achieve health equity in referral rates. Clinician bias as well as lack of education about disparities continue to play a prominent role.
3. Large disparities in the ability to complete the evaluation process affect women, racial/ethnic minority patients, the geographically isolated, and patients who are uninsured or covered by public insurance. This area of the transplantation process remains vague, with only two clinical trials conducted to date and no evidence that interventions have a significant effect on waitlisting rates.
4. Access to living donors represents an unattainable goal for many. Living donor liver transplant is a highly specialized aspect of care and varies significantly from center to center. The adult-to-adult living donor liver transplant consortium investigated safety in living donation, but no group has examined equity. In kidney transplantation, the disparity in the ability of racial/ethnic minority patients to undergo LDKT appears to be in part, but not completely, due to lack of knowledge about the benefits of living donor transplant and particularly preemptive transplant. Educational interventions and culturally-competent and tailored engagement with the transplant center has shown great promise, with several clinical trials still underway.
5. Even among patients who successfully gain access to the transplant waitlist, disparities continue to negatively affect access to high-quality organs. Removal from the waitlist, waitlist mortality, and quality of organ selection all remain inequitable. Though beyond the scope of our review, our search identified more than 200 publications reporting disparities in post-transplant outcomes. This area deserves continued investigation.
6. Social determinants of health are frequently limited to race, gender and insurance status. These variables are collected in national registries, but fail to fully capture the influence of social risk on overall success as a transplant candidate. There is chronic under-utilization of more enhanced social determinants of health, such as food insecurity, educational access and built environment. Despite numerous studies describing observed differences in access to and outcomes after transplant, no work has reliably established causal mechanisms for these differences. Without this, efforts to eliminate these differences will likely continue to have limited success, underscoring the need for improved data collection in this area.

Several limitations warrant mention. First, our review only included studies published in the US and therefore may have omitted relevant studies of social determinants of health and access to transplantation. Similarly, we did not include studies in languages other than English due to lack of resources. Finally, we did not conduct risk of bias assessment of included studies or meta-analysis of findings as the purpose of this scoping review was to collate a wide range of studies of disparities and inequities in access to transplant of all organ types in order to assess the scope of the evidence base to date and identify gaps in evidence.

## Conclusion

In summary, there is a need for improved research in health equity in transplantation. Although the clinical presentation and subspecialty evaluation differ between various subsets of patients with end-stage organ disease, the process of prompt diagnosis, access to subspecialty care, and efficient referral is universal. Equity in access to organ transplantation requires the ability of patients to benefit from early diagnosis of end-stage organ disease and receipt of prompt referral for transplant evaluation. Although patient interest in transplantation plays a role, other potential drivers include patients’ preferences, knowledge of transplantation, challenges navigating the referral and selection processes, communication and trust between patients and clinicians, and structural and personal bias. Very little work has been done to identify and mitigate disparities at this stage in the process and should be the focus of future research to elucidate potential approaches for intervention. Ultimately, an equitable system would allow all patients who have an end organ condition that can be helped by transplant the same access to transplant. In addition to this, the change would ideally be “effortless” to patients so that people, who are already burdened with disease, do not have to make any additional effort to recognize the benefit of this change.

## Supplementary Information

**Additional file 1.** Search strategy report for socioeconomic disparities and organ transplantation in queried databases.

## Data Availability

Not applicable.

## Abbreviations

ACTS: About Choices in Transplantation and Sharing
ADL: Activities of daily living
ASCENT: Allocation System Changes for Equity in Kidney Transplantation
CKD-EPI: Chronic Kidney Disease Epidemiology Collaboration
eGFR: Estimated glomerular filtration rate
ELITE: Enhancing Living Donor Kidney Transplant Education
ESRD: End-stage renal disease
GFR: Glomerular filtration rate
HKTP: Hispanic Kidney Transplant Program
KAS: Kidney allocation system
KTFT: Kidney Transplant Fast Track
LDKT: Living donor kidney transplant
LDLT: Living donor liver transplant
LVAD: Left ventricular assist device
MELD: Model for End-Stage Liver Disease
PREPARED: Providing Resources to Enhance African American Patients’ Readiness to Make Decisions about Kidney Disease
RaDIANT: Reducing Disparities in Access to kidNey Transplantation
SAI: Social Adaptability Index
SES: Socioeconomic status
SPKT: Simultaneous pancreas-kidney transplants
SRTR: Scientific Registry of Transplant Recipients
TALK: Talking About Live Kidney Donation
TALKS: Talking about Living Kidney Donation Support
UNOS: United Network for Organ Sharing
US: United States
USRDS: United States Renal Data System
VA: Veterans Administration
