# Supplementary material for: A scoping review of inequities in access to organ transplant in the United States
Source: Int J Equity Health. 2022 Feb 12;21:22. doi: 10.1186/s12939-021-01616-x (PMC8841123; doi:10.1186/s12939-021-01616-x)
Supplement: Supplementary file 1 — Additional file 1. Search strategy report for socioeconomic disparities and organ transplantation in queried databases. [file 12939_2021_1616_MOESM1_ESM.docx]

**SDC 1.** Search strategy report for socioeconomic disparities and organ transplantation in queried databases

1. MEDLINE (via PubMed)

| Set # |  | Results |
| --- | --- | --- |
| 1  SES | "Ethnic Groups"[Mesh] OR "Minority Health"[Mesh] OR "Race Factors"[Mesh] OR "Hispanic Americans"[Mesh] OR "Mexican Americans"[Mesh] OR "African Americans"[Mesh] OR "Vulnerable Populations"[Mesh] OR "Healthcare Disparities"[Mesh] OR "Health Status Disparities"[Mesh] OR "Socioeconomic Factors"[Mesh] OR "Social Class"[Mesh] OR "Cost of Illness"[Mesh] OR "Poverty"[Mesh] OR "vulnerable population"[tiab] OR "vulnerable populations"[tiab] OR "sensitive population"[tiab] OR "sensitive populations"[tiab] OR underserved[tiab] OR underserving[tiab] OR underserve[tiab] OR disadvantaged[tiab] OR marginalized[tiab] OR marginalised[tiab] OR marginalization[tiab] OR marginalisation[tiab] OR disparities[tiab] OR disparity[tiab] OR inequality[tiab] OR inequalities[tiab] OR "economic burden"[tiab] OR "financial burden"[tiab] OR "travel burden"[tiab] OR "cost of illness"[tiab] OR "cost of sickness"[tiab] OR "cost of disease"[tiab] OR socioeconomic[tiab] OR SES[tiab] OR poor[tiab] OR "low income"[tiab] OR low-income[tiab] OR poverty[tiab] OR impoverished[tiab] OR "low resource"[tiab] OR low-resource[tiab] OR Hispanic[tiab] OR Mexican[tiab] OR Hispanics[tiab] OR Mexicans[tiab] OR Cuban[tiab] OR Cubans[tiab] OR Latin[tiab] OR Latina[tiab] OR Latinas[tiab] OR Latino[tiab] OR Latinos[tiab] OR Latinx[tiab] OR "Spanish speaking"[tiab] OR "Spanish-speaking"[tiab] OR "Spanish speakers"[tiab] OR "Spanish-speakers"[tiab] OR (Spanish[tiab] AND (speaking[tiab] OR language[tiab])) OR "Puerto Rico"[tiab] OR "Puerto Rican"[tiab] OR "Puerto Ricans"[tiab] OR black[tiab] OR blacks[tiab] OR "African-American"[tiab] OR "African American"[tiab] OR "African-Americans"[tiab] OR "African Americans"[tiab] OR racial[tiab] OR ethnic[tiab] OR ethnicity[tiab] OR ethnicities[tiab] OR minority[tiab] OR minorities[tiab] | 1506786 |
| 2  Organ disease | "Liver Failure"[Mesh] OR "End Stage Liver Disease"[Mesh] OR "Kidney Failure, Chronic"[Mesh] OR "Heart Failure"[Mesh] OR ((organ[tiab] OR organs[tiab] OR liver[tiab] OR kidney[tiab] OR renal[tiab] OR heart[tiab] OR pulmonary[tiab] OR lung[tiab] OR pancreas[tiab] OR pancreatic[tiab]) AND (failure[tiab] OR failed[tiab] OR fail[tiab] OR failing[tiab])) OR (("end stage"[tiab] OR "end of life"[tiab] OR EOL[tiab] OR terminal[tiab]) AND (organ[tiab] OR organs[tiab] OR liver[tiab] OR kidney[tiab] OR renal[tiab] OR heart[tiab] OR pulmonary[tiab] OR lung[tiab] OR pancreas[tiab] OR pancreatic[tiab]) AND (disease[tiab] OR diseases[tiab])) | 546196 |
| 3  Transplant | "Transplant Recipients"[Mesh] OR "Organ Transplantation"[Mesh] OR "Living Donors"[Mesh] OR "Tissue and Organ Procurement"[Mesh] OR transplant[tiab] OR transplantation[tiab] OR transplants[tiab] OR transplantations[tiab] OR transplanted[tiab] OR transplanting[tiab] | 500391 |
| 4  Care | "Delivery of Health Care"[Mesh] OR "Waiting Lists"[Mesh] OR "Practice Patterns, Physicians'"[Mesh] OR referral[tiab] OR referrals[tiab] OR refer[tiab] OR referral[tiab] OR referrals[tiab] OR referred[tiab] OR referring[tiab] OR waitlist[tiab] OR waitlisted[tiab] OR waitlisting[tiab] OR waitlists[tiab] OR receipt[tiab] OR recipient[tiab] OR recipients[tiab] OR consultation[tiab] OR consultations[tiab] OR consult[tiab] OR consults[tiab] OR consulted[tiab] OR donor[tiab] OR donors[tiab] | 1793324 |
| 5 | 1 AND 2 AND 3 AND 4 | 4112 |
| 6 | 5 NOT (animals[mh] NOT humans[mh]) | 4067 |
| 7 | 6 NOT (Editorial[ptyp] OR "Case Reports"[ptyp] OR Comment[ptyp]) | 3746 |

2. Embase (via Elsevier)

| Set # |  | Results |
| --- | --- | --- |
| 1  SES | 'minority health'/exp OR 'ethnic or racial aspects'/exp OR 'Hispanic'/exp OR 'Mexican'/exp OR 'African American'/exp OR 'vulnerable population'/exp OR 'health care disparity'/exp OR 'socioeconomics'/exp OR 'social class'/exp OR 'cost of illness'/exp OR 'poverty'/exp OR "vulnerable population":ti,ab OR "vulnerable populations":ti,ab OR "sensitive population":ti,ab OR "sensitive populations":ti,ab OR underserved:ti,ab OR underserving:ti,ab OR underserve:ti,ab OR disadvantaged:ti,ab OR marginalized:ti,ab OR marginalised:ti,ab OR marginalization:ti,ab OR marginalisation:ti,ab OR disparities:ti,ab OR disparity:ti,ab OR inequality:ti,ab OR inequalities:ti,ab OR "economic burden":ti,ab OR "financial burden":ti,ab OR "travel burden":ti,ab OR "cost of illness":ti,ab OR "cost of sickness":ti,ab OR "cost of disease":ti,ab OR socioeconomic:ti,ab OR SES:ti,ab OR poor:ti,ab OR "low income":ti,ab OR low-income:ti,ab OR poverty:ti,ab OR impoverished:ti,ab OR "low resource":ti,ab OR low-resource:ti,ab OR Hispanic:ti,ab OR Mexican:ti,ab OR Hispanics:ti,ab OR Mexicans:ti,ab OR Cuban:ti,ab OR Cubans:ti,ab OR Latin:ti,ab OR Latina:ti,ab OR Latinas:ti,ab OR Latino:ti,ab OR Latinos:ti,ab OR Latinx:ti,ab OR 'Spanish speaking':ti,ab OR 'Spanish-speaking':ti,ab OR 'Spanish speakers':ti,ab OR 'Spanish-speakers':ti,ab OR (Spanish:ti,ab AND (speaking:ti,ab OR language:ti,ab)) OR 'Puerto Rico':ti,ab OR 'Puerto Rican':ti,ab OR 'Puerto Ricans':ti,ab OR black:ti,ab OR blacks:ti,ab OR 'African-American':ti,ab OR 'African American':ti,ab OR 'African-Americans':ti,ab OR 'African Americans':ti,ab OR racial:ti,ab OR ethnic:ti,ab OR ethnicity:ti,ab OR ethnicities:ti,ab OR minority:ti,ab OR minorities:ti,ab | 1954131 |
| 2  Organ disease | 'liver failure'/de OR 'acute liver failure'/exp OR 'acute on chronic liver failure'/exp OR 'chronic liver failure'/exp OR 'end stage liver disease'/exp OR 'kidney failure'/de OR 'acute kidney failure'/exp OR 'chronic kidney failure'/exp OR 'end stage renal disease'/exp OR 'heart failure'/de OR 'acute heart failure'/exp OR 'congestive heart failure'/exp OR 'experimental heart failure'/exp OR 'forward heart failure'/exp OR 'heart ventricle overload'/exp OR 'high output heart failure'/exp OR ((organ:ti,ab OR organs:ti,ab OR liver:ti,ab OR kidney:ti,ab OR renal:ti,ab OR heart:ti,ab OR pulmonary:ti,ab OR lung:ti,ab OR pancreas:ti,ab OR pancreatic:ti,ab) AND (failure:ti,ab OR failed:ti,ab OR fail:ti,ab OR failing:ti,ab)) OR (("end stage":ti,ab OR "end of life":ti,ab OR EOL:ti,ab OR terminal:ti,ab) AND (organ:ti,ab OR organs:ti,ab OR liver:ti,ab OR kidney:ti,ab OR renal:ti,ab OR heart:ti,ab OR pulmonary:ti,ab OR lung:ti,ab OR pancreas:ti,ab OR pancreatic:ti,ab) AND (disease:ti,ab OR diseases:ti,ab)) | 1055159 |
| 3  Transplant | 'graft recipient'/exp OR 'organ transplantation'/exp OR 'living donor'/exp OR transplant:ti,ab OR transplantation:ti,ab OR transplants:ti,ab OR transplantations:ti,ab OR transplanted:ti,ab OR transplanting:ti,ab | 763306 |
| 4  Care | 'health care delivery'/de OR 'clinical practice'/de OR referral:ti,ab OR referrals:ti,ab OR refer:ti,ab OR referral:ti,ab OR referrals:ti,ab OR referred:ti,ab OR referring:ti,ab OR waitlist:ti,ab OR waitlisted:ti,ab OR waitlisting:ti,ab OR waitlists:ti,ab OR receipt:ti,ab OR recipient:ti,ab OR recipients:ti,ab OR consultation:ti,ab OR consultations:ti,ab OR consult:ti,ab OR consults:ti,ab OR consulted:ti,ab OR donor:ti,ab OR donors:ti,ab | 1639640 |
| 5 | 1 AND 2 AND 3 AND 4 | 8064 |
| 6 | 5 AND [humans]/lim | 7302 |
| 7 | 6 AND ([article]/lim OR [article in press]/lim OR [letter]/lim OR [review]/lim) | 3811 |

3. CINAHL (via Ebsco)

| Set # |  | Results |
| --- | --- | --- |
| 1  SES | (MH "Hispanics") OR (MH "Blacks") OR (MH "Race Factors") OR (MH "Special Populations") OR (MH "Healthcare Disparities") OR (MH "Health Status Disparities") OR (MH "Socioeconomic Factors+") OR (MH "Social Class+") OR (MH "Economic Aspects of Illness") OR (MH "Poverty+") OR TI ( "vulnerable population" OR "vulnerable populations" OR "sensitive population" OR "sensitive populations" OR underserved OR underserving OR underserve OR disadvantaged OR marginalized OR marginalised OR marginalization OR marginalisation OR disparities OR disparity OR inequality OR inequalities OR "economic burden" OR "financial burden" OR "travel burden" OR "cost of illness" OR "cost of sickness" OR "cost of disease" OR socioeconomic OR SES OR poor OR "low income" OR low-income OR poverty OR impoverished OR "low resource" OR low-resource ) OR AB ( "vulnerable population" OR "vulnerable populations" OR "sensitive population" OR "sensitive populations" OR underserved OR underserving OR underserve OR disadvantaged OR marginalized OR marginalised OR marginalization OR marginalisation OR disparities OR disparity OR inequality OR inequalities OR "economic burden" OR "financial burden" OR "travel burden" OR "cost of illness" OR "cost of sickness" OR "cost of disease" OR socioeconomic OR SES OR poor OR "low income" OR low-income OR poverty OR impoverished OR "low resource" OR low-resource OR Hispanic OR Mexican OR Hispanics OR Mexicans OR Cuban OR Cubans OR Latin OR Latina OR Latinas OR Latino OR Latinos OR Latinx OR "Spanish speaking" OR "Spanish-speaking" OR "Spanish speakers" OR "Spanish-speakers" OR (Spanish AND (speaking OR language)) OR "Puerto Rico" OR "Puerto Rican" OR "Puerto Ricans" OR black OR blacks OR "African-American" OR "African American" OR "African-Americans" OR "African Americans" OR racial OR ethnic OR ethnicity OR ethnicities OR minority OR minorities) | 599678 |
| 2  Organ disease | (MH "Liver Failure+") OR (MH "Kidney Failure, Chronic+") OR (MH "Heart Failure+") OR TI ( ((organ OR organs OR liver OR kidney OR renal OR heart OR pulmonary OR lung OR pancreas OR pancreatic) AND (failure OR failed OR fail OR failing)) OR (("end stage" OR "end of life" OR EOL OR terminal) AND (organ OR organs OR liver OR kidney OR renal OR heart OR pulmonary OR lung OR pancreas OR pancreatic) AND (disease OR diseases)) ) OR AB ( ((organ OR organs OR liver OR kidney OR renal OR heart OR pulmonary OR lung OR pancreas OR pancreatic) AND (failure OR failed OR fail OR failing)) OR (("end stage" OR "end of life" OR EOL OR terminal) AND (organ OR organs OR liver OR kidney OR renal OR heart OR pulmonary OR lung OR pancreas OR pancreatic) AND (disease OR diseases)) ) | 111646 |
| 3  Transplant | (MH "Transplant Recipients") OR (MH "Organ Transplantation+") OR (MH "Living Donors") OR (MH "Transplant Donors+") OR (MH "Organ Procurement+") OR TI ( transplant OR transplantation OR transplants OR transplantations OR transplanted OR transplanting ) OR AB ( transplant OR transplantation OR transplants OR transplantations OR transplanted OR transplanting ) | 67274 |
| 4  Care | (MH "Health Care Delivery+") OR (MH "Waiting Lists") OR (MH "Practice Patterns") OR TI ( referral OR referrals OR refer OR referral OR referrals OR referred OR referring OR waitlist OR waitlisted OR waitlisting OR waitlists OR receipt OR recipient OR recipients OR consultation OR consultations OR consult OR consults OR consulted OR donor OR donors ) OR AB ( referral OR referrals OR refer OR referral OR referrals OR referred OR referring OR waitlist OR waitlisted OR waitlisting OR waitlists OR receipt OR recipient OR recipients OR consultation OR consultations OR consult OR consults OR consulted OR donor OR donors ) | 471590 |
| 5 | 1 AND 2 AND 3 AND 4 | 590 |

4. Web of Science (via Clarivate)

| Set # |  | Results |
| --- | --- | --- |
| 1  SES | TS=("vulnerable population" OR "vulnerable populations" OR "sensitive population" OR "sensitive populations" OR underserved OR underserving OR underserve OR disadvantaged OR marginalized OR marginalised OR marginalization OR marginalisation OR disparities OR disparity OR inequality OR inequalities OR "economic burden" OR "financial burden" OR "travel burden" OR "cost of illness" OR "cost of sickness" OR "cost of disease" OR socioeconomic OR SES OR poor OR "low income" OR low-income OR poverty OR impoverished OR "low resource" OR low-resource) OR TI=("vulnerable population" OR "vulnerable populations" OR "sensitive population" OR "sensitive populations" OR underserved OR underserving OR underserve OR disadvantaged OR marginalized OR marginalised OR marginalization OR marginalisation OR disparities OR disparity OR inequality OR inequalities OR "economic burden" OR "financial burden" OR "travel burden" OR "cost of illness" OR "cost of sickness" OR "cost of disease" OR socioeconomic OR SES OR poor OR "low income" OR low-income OR poverty OR impoverished OR "low resource" OR low-resource OR Hispanic OR Mexican OR Hispanics OR Mexicans OR Cuban OR Cubans OR Latin OR Latina OR Latinas OR Latino OR Latinos OR Latinx OR "Spanish speaking" OR "Spanish-speaking" OR "Spanish speakers" OR "Spanish-speakers" OR (Spanish AND (speaking OR language)) OR "Puerto Rico" OR "Puerto Rican" OR "Puerto Ricans" OR black OR blacks OR "African-American" OR "African American" OR "African-Americans" OR "African Americans" OR racial OR ethnic OR ethnicity OR ethnicities OR minority OR minorities) | 1,924243 |
| 2  Organ disease | TS=(((organ OR organs OR liver OR kidney OR renal OR heart OR pulmonary OR lung OR pancreas OR pancreatic) AND (failure OR failed OR fail OR failing)) OR (("end stage" OR "end of life" OR EOL OR terminal) AND (organ OR organs OR liver OR kidney OR renal OR heart OR pulmonary OR lung OR pancreas OR pancreatic) AND (disease OR diseases)) ) OR TI=(((organ OR organs OR liver OR kidney OR renal OR heart OR pulmonary OR lung OR pancreas OR pancreatic) AND (failure OR failed OR fail OR failing)) OR (("end stage" OR "end of life" OR EOL OR terminal) AND (organ OR organs OR liver OR kidney OR renal OR heart OR pulmonary OR lung OR pancreas OR pancreatic) AND (disease OR diseases))) | 573195 |
| 3  Transplant | TS=(transplant OR transplantation OR transplants OR transplantations OR transplanted OR transplanting) OR TI=(transplant OR transplantation OR transplants OR transplantations OR transplanted OR transplanting) | 648339 |
| 4  Care | TS=(referral OR referrals OR refer OR referral OR referrals OR referred OR referring OR waitlist OR waitlisted OR waitlisting OR waitlists OR receipt OR recipient OR recipients OR consultation OR consultations OR consult OR consults OR consulted OR donor OR donors) OR TI=(referral OR referrals OR refer OR referral OR referrals OR referred OR referring OR waitlist OR waitlisted OR waitlisting OR waitlists OR receipt OR recipient OR recipients OR consultation OR consultations OR consult OR consults OR consulted OR donor OR donors) | 1192148 |
| 5 | 1 AND 2 AND 3 AND 4 | 3045 |
| 6 | 5 *AND***DOCUMENT TYPES:** (Article OR Letter OR Review) | 2,973 |
